# Seeing for speaking: Semantic and lexical information provided by briefly presented, naturalistic action scenes

**DOI:** 10.1371/journal.pone.0194762

**Published:** 2018-04-13

**Authors:** Pienie Zwitserlood, Jens Bölte, Reinhild Hofmann, Claudine C. Meier, Christian Dobel

**Affiliations:** 1 Institute for Psychology, University of Münster, Münster, Germany; 2 Otto-Creutzfeldt Center for Cognitive Neuroscience, University of Münster, Münster, Germany; 3 Clinic for Phoniatrics and Pediatric Audiology, University of Münster, Münster, Germany; 4 Department of Otorhinolaryngology, Medical Faculty, University of Jena, Jena, Germany; Utrecht University, NETHERLANDS

## Abstract

At the interface between scene perception and speech production, we investigated how rapidly action scenes can activate semantic and lexical information. Experiment 1 examined how complex action-scene primes, presented for 150 ms, 100 ms, or 50 ms and subsequently masked, influenced the speed with which immediately following action-picture targets are named. Prime and target actions were either identical, showed the same action with different actors and environments, or were unrelated. Relative to unrelated primes, identical and same-action primes facilitated naming the target action, even when presented for 50 ms. In Experiment 2, neutral primes assessed the direction of effects. Identical and same-action scenes induced facilitation but unrelated actions induced interference. In Experiment 3, written verbs were used as targets for naming, preceded by action primes. When target verbs denoted the prime action, clear facilitation was obtained. In contrast, interference was observed when target verbs were phonologically similar, but otherwise unrelated, to the names of prime actions. This is clear evidence for word-form activation by masked action scenes. Masked action pictures thus provide conceptual information that is detailed enough to facilitate apprehension and naming of immediately following scenes. Masked actions even activate their word-form information–as is evident when targets are words. We thus show how language production can be primed with briefly flashed masked action scenes, in answer to long-standing questions in scene processing.

## Introduction

Humans perceive and talk about complex events all the time. They are able to extract information from complex visual scenes very rapidly, even when these are only briefly seen–as in zapping through TV channels. The overall meaningfulness or coherence of scenes, as well as their gist, involving a coarse understanding and categorization of the scene as a whole (a park, a track course), can be apprehended within a glance [1, 2, 3]. Moreover, decisions as to whether or not a scene includes a building, a vehicle, an animal, can be made with high accuracy even with very briefly flashed scenes [4, 5].

One driving question concerns the nature and detail of the representations that underlie this amazing performance. Whereas scene coherence might well be signalled by global visual information [1], extracting the scene’s gist and detection of particular scene elements seem to require semantic information. There is ample evidence that this can be done within a first glance, but researchers disagree on what exactly can be extracted during early visual processing. Earlier studies propose that basic-level representations (e.g. dog) can be accessed faster than superordinate (e.g. animal) levels [6, 7], but more recent evidence points to the opposite ([8, 9], for an overview see [10]). Wu and colleagues argue that early visual processing results in a rather coarse representation, allowing for superordinate categorization, whereas detailed within-category differentiation requires additional visual analysis. Potter and colleagues have a different view on this. They demonstrated that presentation durations as short as 13 ms suffice to access semantic and conceptual information, such that conscious detection of highly complex visual information is possible [11, 12, 13]. The divergence in results and interpretations might stem from the different tasks used, which capture different aspects of fast visual processing and of additional memory processes. For example, Wu and co-authors [10] employed forced-choice saccadic tasks, in which the relevant response (“look at the picture that contains an animal”) is made within 150 ms or so, while pictures remained on screen. Potter and co-authors [11, 12] used rapid visual serial presentation, with series of briefly flashed pictures, but responses were made after the final stimulus (“were there flowers in one of the pictures?”). Thus, whereas the first task does not require memory involvement, the second one depends on it. Obviously, processing does not come to a halt when the stimulus is no longer visible, and further processing involves memory. This division of labour is supported by evidence for separate brain networks for visual-feature and memory aspects of scene processing [14].

Note that both groups [10, 11] agree that a first sweep of feed-forward processing can account for their results, and that re-entrant processing from higher cognitive / neural processing levels is not necessary. Crouzet and colleagues even hypothesized that such fast feed-forward processes are hardwired in the visual system, at least for categories such as animals [15]. Taken together, there is agreement that brief presentation suffices to process highly complex pictures without additional attention shifts. It is not yet clear what levels or representations can be accessed on the basis of such information, and the answer to this question strongly depends on the task employed.

In contrast to such saccadic and decision tasks, humans need more than global categorization or specific category information when they talk about events and scenes—something they do all the time. Full scene detail and semantic access is needed, as well as the words that describe the scene elements: names for objects, and verbs that denote actions. Knowing about the presence or absence of a vehicle or an animal does not suffice: to decide between “giving” and “showing”, or “pushing” and “pulling”, the details of a scene must be identified before the lexical information, the words denoting the objects or actions, can be reliably selected. As such, naming of objects and complex scenes in particular recommends itself as a valuable tool to investigate higher-order perceptual processes. It is at this interface between visual perception and speaking that the present research is situated.

The research reported here focuses on a particular type of scene, namely actions. We investigated under which conditions conceptual-semantic details concerning the action become available and can facilitate speech production–the naming of the action. We used a priming paradigm with action scenes as primes, briefly presented and subsequently masked, and action scenes or action words (verbs) as targets. Before describing the details of the experiments and the predictions, we briefly review relevant research and theory on the processing of (action) scenes, on priming with scene pictures, and on picture and word naming, the two tasks used in our study.

### Perceiving action scenes and naming actions

The amazing ability to categorize scenes into broad categories, demonstrated by Thorpe and colleagues [16, 17] may well be based on global scene characteristics such as spatial layout, rather than on actual identification ([1, 18] for a model. [19] for an excellent overview). There is some indication that access to superordinate information (“man-made” vs. “nature-made”) precedes basic-level information (“street”, “forest”), which again precedes perception of a particular action [20, 21]. For the present purposes, this implies that the overall spatial layout of related and unrelated scenes used as primes should be controlled.

Given that action scenes were only briefly presented as primes, we used pictures of action scenes instead of movies, in which the action unfolds over time [22]. What can be extracted from such action-scene pictures? With briefly presented action scenes, Dobel, Gumnior, Bölte and Zwitserlood [23] manipulated scene coherence (coherent: Indian shooting a buffalo; incoherent: Indian and buffalo both facing outwards). Coherence was judged correctly in 80% of the cases when actions were presented for 100 milliseconds. Glanemann et al. [1] showed that even 30 milliseconds suffice for this type of decision, when naturalistic photos are used instead of line drawings. Perception of scene coherence thus seems to have a similar time course as the apprehension of scene gist. But what about the timing with which scene elements, such as objects or actors, can reliably be identified? As mentioned above, objects may be detected even peripherally when it is known what to look for—a given in categorization tasks. Without such prior knowledge, potentially interesting scene elements are fixated when fixations are allowed, which of course applies to normal vision. These fixations are guided by image properties that can be perceived before sending the eyes into the scene, rather than by top-down factors such as task demands [19].

A study in which fixations into action scenes were not only allowed but used as dependent variable is reported by Griffin and Bock [24]. Using line drawing of actions (e.g. a girl shooting a man), participants had to freely describe scenes or, in a different condition, to send their eyes to the patient of the action (patient detection) as quickly as possible after picture onset. In the latter task, fixations of the patients began to diverge from fixation of the agent about 300 ms after picture onset. Griffin and Bock argued that their data support very early ideas on sentence production formulated by Wilhelm Wundt, who proposed that initial apprehension precedes formulation [25]. This entails that scenes must be first at least coarsely encoded before the initiation of an utterance can start.

But eye movements into the picture are not even necessary for correct naming. In the study by Dobel et al. [23], agents and patients could be correctly named well above chance level when scenes were presented for a mere 100 milliseconds, and masking rendered effective eye-movements into the scenes unlikely. Note that actions could not be named correctly with this brief presentation duration. Glanemann [26] observed that a presentation of 150 ms sufficed to identify the patient (98% correct) merely through peripheral viewing. Actions could be named correctly only if their identity could be extracted from the global scene layout (“e.g., “kicking”, with an outstretched leg), not when details about objects (e.g., a pen, for “writing”) were needed. This clearly shows that fixations are not necessary for the identification of scene elements, not even for their naming (see [27] for an overview). Hafri, Papafragou, & Trueswell [28] showed that event roles could be identified upon masked presentation of naturalistic action scenes, shown for 37 or 73 ms. Importantly, and as in Dobel et al. [27], role identification with short presentation depended on physical features, such as outstretched arms or legs, that are typical for agenthood. Similarly, detecting the coherence of scenes with brief presentation durations (20, 30, 50, 100 ms) strongly depended on the overall ‘Gestalt’ of interacting agents. Even when scenes were presented for 100 ms, action coherence could not be judged correctly when the objects involved in the action determined coherence (e.g., “shooting” someone with a hand brush; [1]). We argued there that, at least for actions involving two agents, presentation durations of 100 ms or below do not suffice to provide intentional access to visual details or semantic representations. As such, this result confirms the conclusion of Wu and colleagues [10] that the visual system can rapidly access coarse representations, but that additional analysis is needed for detailed categorization.

In our study, we concentrated on actions, not on objects or actors involved in the action. Instead of detection or role identification, we used action naming. Naming elements of scenes, or actions, is impossible without their identification, and goes well beyond role identification. Our study is relevant for two different theoretical conceptions about scene processing. On the one hand, there is the rapid uptake of information from complex (action) scenes, as studied in the context of theories of scene processing [11, 16]. Any impact of briefly glimpsed prime actions is informative about these aspects of scene processing. On the other hand, scene apprehension is studied in speech production, as the first step towards correct scene description [24, 29]. Here, scene processing involves a series of fixations on relevant scene details that are tightly coupled with parts of the utterance–the scene description. Using masked action scenes as primes, we address the impact of the first aspect, the information gained from briefly glimpsed scenes, on the second process, the naming of target scenes that are available for detailed inspection before naming. Note that we were not interested in whether or not prime scenes reached consciousness. We merely wished to constrain information uptake from the outside event, the prime, to a period that prevents visual inspection, by means of eye movements. Of course, internal processing is not prevented by a visual mask, and it is this processing that we are interested in.

Briefly presented action scenes may provide visual, pictorial and conceptual information [11, 12], and our prime conditions allow us to distinguish between these aspects. Moreover, briefly flashed scenes may also provide lexical information, for example, the name of the action. In models of speech production, conceptual information precedes lexical information: speakers need to decide what to talk about before retrieving word forms associated with the concepts–their “names”. In terms of scene perception and naming: Scene elements (objects, actors, action) need to be identified before lexical information (word forms, phonemes, and articulatory information) is activated [30]. The naming of individually presented objects minimally takes 600 ms from picture onset [31]; naming elements of action scenes takes longer [32, 33]. Thus, naming latencies are not a fruitful measure to investigate what information is available early on during scene processing. Our study used a different methodology: the priming paradigm. We examined the impact of briefly presented action scenes on the processing and naming of subsequently presented action scenes, or action verbs.

If the naming of an action picture is speeded by the presence of a similar action presented as prime (as in experiment 1 and 2), facilitation might be due to shared visual, conceptual, and lexical information. We controlled the first by a high visual similarity between related and unrelated action primes, so that conceptual and lexical information remain. There is some evidence from picture-picture studies and from studies using the visual-world paradigm that task-irrelevant pictures activate their names. For example, Morsella and Miozzo [34] showed two superimposed line drawings of objects, one in red, one in green, and participants only named the objects of one colour (targets) while ignoring the others (distractors). Naming was faster when the names of target and distractor shared their word-onset phonemes (e.g. bell-bed) than when they were unrelated (bell-hat). Apparently, the distractor’s name was automatically activated, and facilitated picture naming when their word forms were similar. These results were replicated by Naverette and Costa [35], Meyer and Damian [36] and Damian and Dumay [37]. McQueen and Huettig [38] showed that fully visible pictures interfered with lexical decisions on subsequent spoken words, when picture name and word were form-related (picture of a foot, word “fool”). Similarly, Chabal and Marian [39], with the visual-world paradigm, showed that objects available for scrutiny in a search display activate their names, even though no speech production is involved in the task.

The studies mentioned above investigated phonological access from pictures onto the naming of, or decision on, pictures with related names, with ample time for the processing of these pictures. In no study, a response to words was required, with one exception. Levelt and colleagues [40] showed pictures that had to be named, but on some trials words were presented briefly after a picture. In that case, picture naming had to be postponed and a lexical decision on the word had to be performed first. Interference resulted if the word was phonologically related to the previous picture. Thus, whether pictures activate their word form, can be measured by using words. This is what we did in Experiment 3. Note that the pictures that caused effects on words in the Levelt et al. study had to be named, even if naming had to be postponed because of the intervening word stimulus. Thus, when in speaking mode, activation of lexical information of picture primes is possible.

### Experimental considerations

Experiments 1–3 were designed to investigate the types of information activated upon the brief exposure to action scenes. We presented naturalistic action photos as primes for 150, 100, or 50 milliseconds. These primes were immediately followed by a mask, to constrain visual information uptake to the duration of prime presentation. Next to their gist and coherence, action pictures may activate conceptual information about the action itself, and, potentially, lexical information–their name. We investigated what information becomes activated by the prime picture, by assessing its effects on target naming. The picture targets from Experiments 1 and 2 were either identical to the primes, different scenes showing the same action, or unrelated to their primes. A neutral prime was included in Experiment 2, to assess the nature (interference, facilitation) of the effects obtained in Experiment 1. Finally, we investigated whether masked action primes activate their word-form information, by using words instead of pictures as targets. The relation between the name of the prime action and the target was either identical (action “to write”, target word *write*) or phonologically related (action “to weep”, target word *sleep*). Given our earlier work [26, 27] we predicted effects of semantic relatedness even for complex action scenes, at least for longer prime durations. For Experiment 3, we predicted facilitation when target verb denotes the action of the prime. This facilitation could be due to word-form activation by the prime scene, or by convergence at the same concept by action scene and target word. This is different in the phonological condition, where there is only form overlap. If masked action scenes activate their word form, these can either facilitate or interfere with the naming of a word target–or both. Facilitation is often observed in word naming when primes and targets share their onset (as in *boaf–boat, or goat–goal; [41]), an effect also seen for masked primes and targets from the two languages of bilinguals [42, 43]. This effect is attributed to the activation, by the prime, of onset phonemes needed in word naming [44]. When prime and target share their rhyme, but have a different onset (as in goat–boat), effects sometimes turn into interference, since the positive effect of shared onsets is absent, and in tasks other than word naming, form-related primes most often interfere with target processing (cf. [40, 44]; for an overview see [45]).

In sum, experiments 1 and 2 tested for semantic effects by masked picture primes, and facilitation of target-picture naming by prime pictures showing the same action would constitute evidence for semantic/conceptual activation. Experiment 3 assessed whether masked picture primes activate lexical information, and any impact–positive or negative—of the mere name relatedness of prime actions and target words constitutes evidence that masked complex action scenes activate their word forms, independent of semantic similarity.

## Experiment 1: Picture-Picture-Priming with three prime durations

In experiment 1, we assessed the processing of complex naturalistic pictures scenes depicting everyday actions. We expected that such action pictures would facilitate the perception and naming of subsequent related pictures, and investigated under which presentation conditions this would be the case. The prime pictures were briefly presented and subsequently masked, and their exposure duration was manipulated. We used naturalistic action photos that, despite their visual complexity, should be easier to process than more stylized pictures or line drawings [46]. Action photos were combined with three different primes. Primes and targets were either identical, showed the same action but with very different stimuli (different actors, background and/or action phases, or different variants of an action, such as breaststroke vs. freestyle, for swimming), or were unrelated.

The stimuli had to fulfil several criteria. First, action prime and target picture in the same-action condition should activate the same concept. We conducted Pretest 1 to ascertain that action photos were identified correctly in >80%, and named consistently. Next, we controlled for effects of overall spatial layout, which can be perceived extremely rapidly [18]. Potential priming effects should not derive from differences between same-action and unrelated primes with respect to their overall spatial similarity to the targets, but to differences in conceptual-semantic information. Pretest 2, with the negatives of the action photos presented blurred, ascertained that the primes in the critical conditions did not differ in their visual layout.

The predictions for the related prime conditions were as follows. Naming should be fastest in the identical condition, given that the prime is visually identical to the target, and thus provides the visual, conceptual and lexical information needed for target naming. The same-action primes provide the same conceptual and lexical information, but with different pictures (different actors, background and layout). If the same-action primes activate semantic information of the action, this should speed up target naming, relative to unrelated primes. To investigate the timing with which semantic information is available, we varied the duration of prime presentation from 150, 100 to 50 ms. Note that primes were always masked, to interrupt visual input.

### Method

This study consisted of three sub-experiments (A-C), with different participants tested within the three prime durations (150 ms, 100 ms, and 50 ms).

#### Participants

A total of 84 participants took part in Experiment 1, all native speakers of German, students from the University of Münster, with normal or corrected-to-normal vision. Participants were recruited between January and November 2008 and gave verbal informed consent to take part. The 24 students that participated in Experiment 1A were aged between 21 and 32 (22 female), the 30 subjects in Experiment 1B were between 20 and 35 years (26 female) and the 30 subjects from Experiment 1C were between 19 and 40 (25 female). Participants provided oral informed consent and received either course credit or were paid for their participation.

#### Stimulus material

A set of 49 actions was pretested for name consistency. Two or three different pictures for each action were available, 113 in total, depicting actors engaged in a meaningful action against different natural backgrounds (e.g., eat, dance, kick, paint, peel). Actors were 14 different persons (6 male, 8 female). The pictures were taken with an *Olympus® Camedia C-5060 Wide Zoom Digital Compact Camera*. All pictures were trimmed and resized to 800 x 800 pixels with *IrfanView* software. In Pretest 1, each of 49 actions (15 with three and 29 with two different photos) was shown for 150 ms, unmasked, on a *Samsung R40* notebook, using *Presentation®* software. Twenty subjects wrote down the name for each stimulus. Actions that were not identified correctly, or with less than 80% naming consistency, were removed from the set (n = 19). For actions with three photos, the two variants with the highest naming accuracy were selected. Of the remaining 30 actions, one variant was used as target throughout.

Targets were combined with three different primes. In the Identical Condition, the prime was the exact same photo as the target. Prime and target in the Same-Action Condition showed the same action, but with different actors, layout and background. In the Unrelated Condition, primes and targets were semantically and phonologically unrelated, had a different layout and often a different action orientation than their target picture. To counterbalance the amount of related prime-target pairs, 30 fillers with unrelated prime-target-pairs were included, amounting to a total of 120 trials. Gender, actor orientation (left, central, right) and left/right-handedness of actors were balanced across pictures, in part by mirroring the pictures.

**Fig 1:**
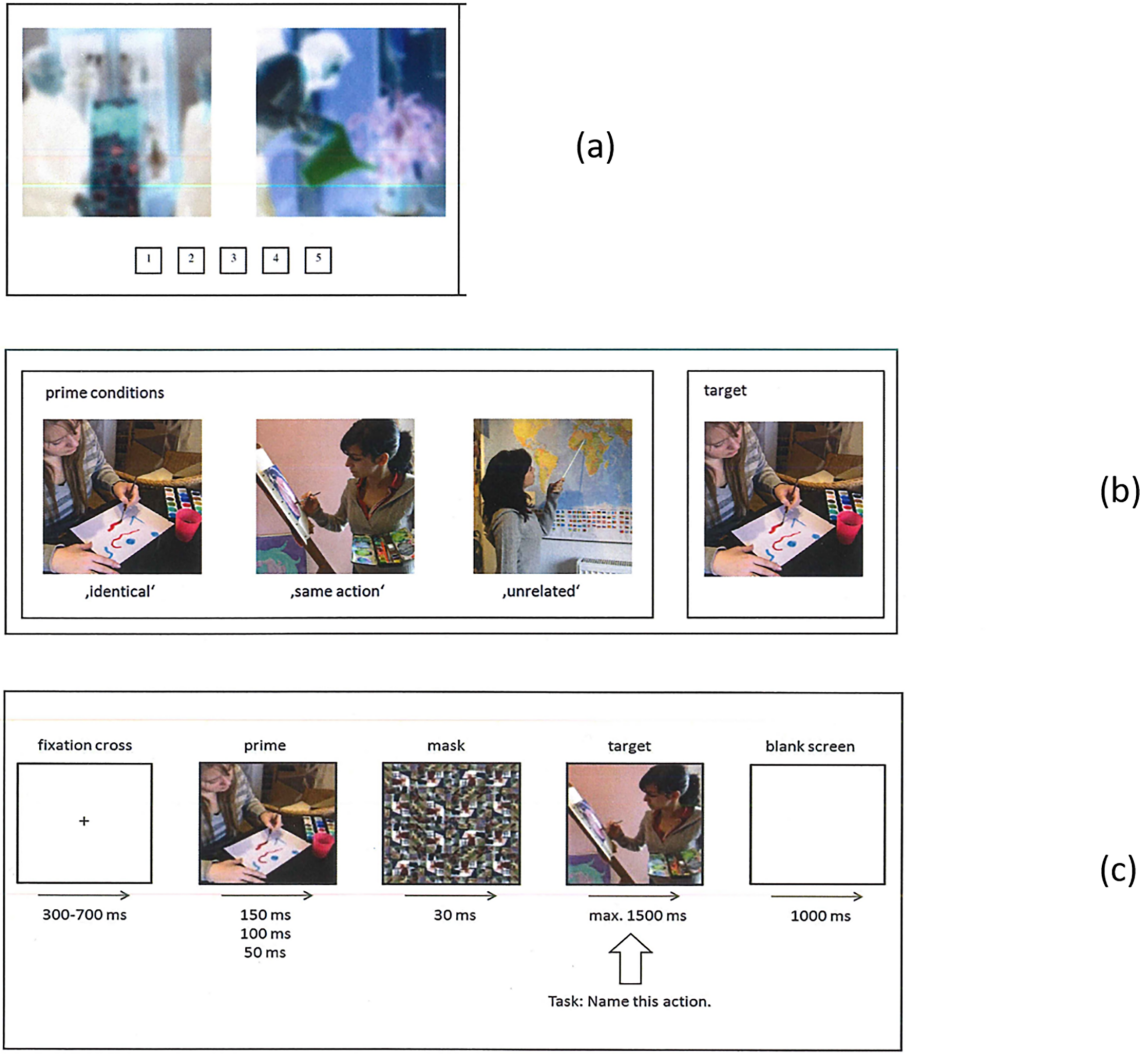
1a-1c: Examples of Gaussian-filtered stimuli for (a) pretest 2, (b) prime types and (c) trial structure.

We also tested whether same-action and unrelated action primes differed in similarity of their spatial layout to the targets [3, 18]. Pictures were transformed into their negative image with *IrfanView*, and blurred by applying a Gaussian blur filter (c = 10), using *Photoshop*. This procedure preserves the overall shape and structural layout, but details become vague. In a paper-pencil test, 20 subjects rated all picture pairs on a five-point Likert Scale with respect to similarity of visual layout, ignoring color (1 = no similarity, 5 = high similarity; see Fig 1A for examples). The Same-Action pictures had mean similarity to the targets of 2.95 (*SD* 0.66), the mean of the Unrelated set was 2.47 (*SD* 0.83). The difference between the two sets was significant (*t*_(29)_ = 2.842, *p* = .008, *r* = .467). After exclusion of six actions, the means for Same-Action (*M* = 2.79, *SD* 0.64), and Unrelated (*M* = 2.64, *SD* = 0.84) no longer differed *t*_(23)_ = 1.049, *p* = .305, *r* = .214. Note that all 30 actions remained in the experiment, but the six actions responsible for the differences were excluded from the analyses.

A mask was created for the primes, consisting of 16 x 16 squares (see Fig 1C for an example). These squares were cut-outs from the experimental photos, but showed no elements of actions. The individuals displayed on the sample stimuli shown in the figures provided written informed consent (as outlined in PLOS consent form) to publish these photographs.

#### Design

Picture targets were repeated three times (Presentation 1–3) in the course of the experiment, distributed over threeblocks with a Latin-square ordering. Each block had a different prime for each target, and equal numbers of primes in each condition. Six experimental lists, each with three blocks, were created, varying the order of blocks. Each block consisted of 40 different action scenes, ten from each prime condition, and ten unrelated fillers (to balance the ratio of related and unrelated trials). The trials in each block were randomized separately for each list. Together with six practice trials, this resulted in 126 prime-target pairs. Each participant received one list, that is, saw each action target three times (within-subject design), once in each of the three prime conditions. Note that repetition of targets is common practice in picture-naming studies, and that three repetitions is not uncommon. Moreover, we checked for repetition effects and potential interactions with prime type. There were thus two factors in the experiment: PRIME TYPE (Identical, Same-Action, and Unrelated) and TARGET REPETITION (Presentation 1–3).

#### Apparatus

The experiment was run by the software ‘*SR Research Experiment Builder*’. The pictures were displayed on a 21-inch *Samsung Syncmaster 1100p* monitor with a screen resolution of 1024 x 768 pixels and a refresh rate of 100 Hz. Naming latencies were measured by voice-key, from the microphone of a *Sennheiser Pro* headset.

#### Procedure

All participants were tested individually. After reading the instructions on the monitor, the room was shaded for the duration of the experiment. The task was to name the action shown in the target picture as quickly as possible using an infinitive German verb form (e.g., “malen”, to paint; “lesen”, to read).

The trial sequence started with a fixation cross in the center of the screen, with jittered duration (700 ms +/- 300 ms). The action prime was presented next, centered, for 150 ms (Exp. 1A), 100 ms (Exp. 1B) or 50 ms (Exp. 1C), immediately followed by a mask presented for 30 ms. The target action appeared immediately after the mask, for a maximal duration of 1500 ms, but disappeared upon speech onset. Answers were checked online for correctness by the experimenter, and errors were registered and coded. The experiment lasted approximately 20 minutes. The experimental procedure of all experiments conforms to the Declaration of Helsinki and has been approved by the Ethics committee of the Department of Psychology and Sport Sciences, U. Münster (nr. 2017–38).

### Results

Data were analyzed using R (3.4.1), RStudio (1.0.143), and the packages ez (Version 4.4.) and apa (0.2.0). Reported here are the results from ANOVAs on subjects and items. For all experiments, a direct comparison of the outcomes of ANOVAs and analyses with linear mixed models (LMM) is possible, because these are included as supplementary materials in S1 File. Coded as incorrect responses were failures to reply, voice-onset errors, noise, self-corrections, hesitations, inconsistent naming over target repetitions, naming errors, and verbs complemented with an object (e.g., correct answer: “lesen”, to read, wrong answer: “Brief lesen”, read letter). Error trials were removed from the data set, so were extreme reaction times (details are provided with each experiment). In addition, six item sets were discarded to equate visual similarity between prime and target pictures (see Pretest 2).

Analyses of variance (ANOVA) with repeated measures, including the within-subject factors PRIME TYPE (Identical, Same-action and Unrelated) and TARGET REPETITION (Presentation 1–3) were performed over subjects and items. In case of significant main effects, prime-type condition means were compared with *t*-tests for paired samples and Bonferroni correction was applied for multiple testing (*p* < .017). The data for Experiment 1A-1C are presented separately, followed by an analysis with Exposure Duration (= Experiment) as factor. The data are shown in Table 1.

**Table 1:** Experiment 1a-1c, mean naming latencies in ms, standard deviations (between brackets), percentage excluded responses, and priming effects as a function of Prime Type and experiment.

	Experiment 1A (150 ms)		Experiment 1B (100 ms)		Experiment 1C (50 ms)	
Prime Type	RT (SD)	*%*	RT (SD)	*%*	RT (SD)	*%*
Identical	589 (95)	10.1%	700 (82)	8.5%	721 (81)	10.8%
Same-Action	654 (92)	9.2%	746 (77)	9.0%	748 (89)	9.6%
Unrelated	746 (95)	12.5%	832 (63)	11.5%	778 (87)	9.4%

#### Experiment 1A (150 ms)

Errors (7.1% of trials) and extreme reaction times (less than 200 ms or above 1220 ms; 3.5%) were discarded from the data (see Table 1). The mean response time was 589 ms, 95% CI[549, 629] with Identical primes, 654 ms, 95% CI[615, 692] with Same-Action primes, and 746 ms, 95% CI[706, 786] with Unrelated primes (see Table 1). The main effects of PRIME TYPE, *F1*_(2, 46)_ = 110.07, *p* < .001, $\eta_{g}^{2}=.27$; *F2*_(2, 46)_ = 79.15, *p* < .001, $\eta_{g}^{2}=.41$ and REPETITION, *F1*_(2, 46)_ = 64.55, *p* < .001, $\eta_{g}^{2}=.24$; *F2*_(2, 46)_ = 122.55, *p* < .001, $\eta_{g}^{2}=.39$ were significant, but did not interact: *F1*_(4, 92)_ = 2.13, *p* = .084, $\eta_{g}^{2}=.01$; *F2*_(4,92)_ = 2.09, p = .088, $\eta_{g}^{2}=.03$ As expected, responses became faster as targets were repeated. Both priming effects were significant (Identical–Unrelated: *t1*_(23)_ = 12.99, *p* < .001, *d* = .260; *t2*_(23)_ = 12.16, *p* < .001, *d* = 2.43; Same-Action–Unrelated: *t1*_(23)_ = 8.34, *p* < .001, *d = 1*.*67*; *t2*_(23)_ = 9.02, *p* < .001, *d* = 1.80), and so was the difference between Identical and Same-Action primes (*t1*_(23)_ = 7.70, *p* < .001, *d =* .*154*; *t2*_(23)_ = 4.23, *p* < .001, *d* = 0.85). Fig A in S2 File illustrates the results for Repetition by Prime Type.

#### Experiment 1B (100 ms)

Errors (5.2%) and extreme reaction times (less than 254 ms or above 1206 ms; 4.4%) were discarded from the data (see Table 1). The mean response time was 700 ms, 95% CI[670, 731] with Identical primes, 746 ms, 95% CI[717, 774] with Same-Action primes, and 832 ms, 95% CI[808, 856] with Unrelated primes. The effects for PRIME TYPE, *F1*_(2, 58)_ = 176.17, *p* < .001, $\eta_{g}^{2}=.29$; *F2*_(2, 46)_ = 100.96, *p* < .001, $\eta_{g}^{2}=.42$ and REPETITION, *F1*_(2, 58)_ = 57.20, *p* < .001, $\eta_{g}^{2}=.20$; *F2*_(2,46)_ = 55.21, *p* < .001, $\eta_{g}^{2}=.33$, were significant, but there was no interaction, *F1* < 1, *F2* < 1. As in Experiment 1A, naming became faster over target repetitions, and all differences between means were significant: Identical and Unrelated, *t1*_(29)_ = 16.65, *p* < .001, *d* = 2.99; *t2*_(23)_ = 11.76, *p* < .001, *d* = 2.35; Same-Action and Unrelated, *t1*_(29)_ = 11.59, *p* < .001, *d* = 2.08; *t2*_(23)_ = 10.16, *p* < .001, *d* = .2.03 and Identical and Same-Action, *t1*_(29)_ = 7.73, *p* < .001, *d* = 1.39; *t2*_(23)_ = 5.4, *p* < .001, *d* = 1.08. Fig B in S2 File shows the data for Repetition by Prime Type.

#### Experiment 1C (50 ms)

Errors (5.4%) and extreme reaction times (less than 200 ms or above 1214 ms; 4.5%) were discarded from the data (see Table 1). The mean response time was 721 ms, 95% CI[691, 751] with Identical primes, 748 ms, 95% CI[715, 781] with Same-Action primes, and 778 ms, 95% CI[745, 810] with Unrelated primes (see Table 1). The main effects for REPETITION, *F1*_(2, 58)_ = 119.94, *p* < .001, $\eta_{g}^{2}=.22$; *F2*_(2,46)_ = 73.33, p < .001, $\eta_{g}^{2}=.99$ and PRIME TYPE, *F1*_(2, 58)_ = 29.72, *p* < .001, $\eta_{g}^{2}=.06$; *F2*_(2,46)_ = 25.16, p < .001, $\eta_{g}^{2}=.08$, were significant, but again, there was no interaction: *F1*_(4, 116)_ = 1.44, *p* = .225, $\eta_{g}^{2}<.01$; *F2* < 1. As with the longer exposure durations, naming became faster over target repetitions, and all prime-condition means differed reliably: Identical and Unrelated, *t1*_(29)_ = 8.57 *p* < .001, *d* = 1.54; *t2*_(23)_ = 6.89, *p* < .001, *d* = 1.38; Same-Action and Unrelated, *t1*_(29)_ = 3.70, *p* < .001, *d* = 0.66; *t2*_(23)_ = 3.47, *p* < .001, *d* = 0.69, and Identical and Same-Action, *t1*_(29)_ = 3.67, *p* = .001, *d* = 0.66; *t2*_(24)_ = 3.71, *p* = .001, *d* = .74. Figure C in S2 File illustrates the results for Repetition by Prime Type.

### Discussion

We investigated whether briefly shown action scenes activated sufficient information to influence the naming of subsequent action targets. Action pictures indeed influenced the naming of the targets, in the direction predicted. Naming was fastest when primes and targets were identical, and slowest when prime and target actions were unrelated. The priming by identical actions was highly significant for all three exposure-durations. In between, but also robust, were effects of primes that show the same action, but with different layout, background, actors, and objects. The speeded naming, in this condition, is most probably due to conceptual-semantic information shared between prime and target. Whether lexical information (the verbal label of the action) is also activated and contributes to target naming was investigated in Experiment 3. Importantly, we observed reliable effects of identical and same-action primes even with the shortest exposure duration. This flash-like presentation of an action provides enough information to influence naming of an immediately following action. This clearly extends research on the rapid detection of certain aspects of complex scenes, such as the presence of animals [4], on the identification of roles in action scenes [28] and on recognition of scenes presented in sequence [12]. It is noteworthy that the effects–in particular of same-action primes—are smaller with brief prime exposure duration (50 ms) than with longer prime durations (100 and 150 ms). This may well be due to the additional processing time for primes with longer durations, but possibly, there are two processes at work: facilitation by identical or related primes, and inhibition by unrelated primes. Given the smaller effects at the shortest prime duration, in terms of the difference between related and unrelated primes, inhibition by unrelated scene primes might be absent because it requires more time than facilitation by related primes. To investigate this possibility, we conducted Experiment 2, with neutral primes.

## Experiment 2: Picture-Picture-Priming with neutral primes

Experiment 2 investigates potential contributions of facilitation, by related picture primes, and interference, by unrelated primes, to the effects observed in Experiment 1. For this purpose, neutral primes were included, consisting of scrambled versions of action pictures. The scrambling removed all semantic content from the pictures.

If the priming effect with the longer presentation durations (100 and 150 ms) is composed of both interference and facilitation, we expect naming latencies after neutral primes to be in between those for related and unrelated primes. We opted for the shortest of these two presentation times: 100 ms. Also different from Experiment 1 was the mirroring of pictures in the Identical condition. This should reduce visual similarity between primes and targets–at least in early visual perception. Note that Biederman and Gerhardstein [47] suggest that object recognition is normally viewpoint-invariant, unless contaminated by the “need to distinguish between mirror reflections of the same object” (p. 1163). Given that orientation is task-irrelevant, we predict priming effects to be similar to those obtained in Experiment 1.

### Method

#### Participants

Thirty native speakers of German (24 female, 20–33 years of age), from the same population as in Experiment I, were recruited between March and June 2009. All participants gave verbal informed consent and received course credit. None had taken part in Experiment I.

#### Material

**Fig 2:**
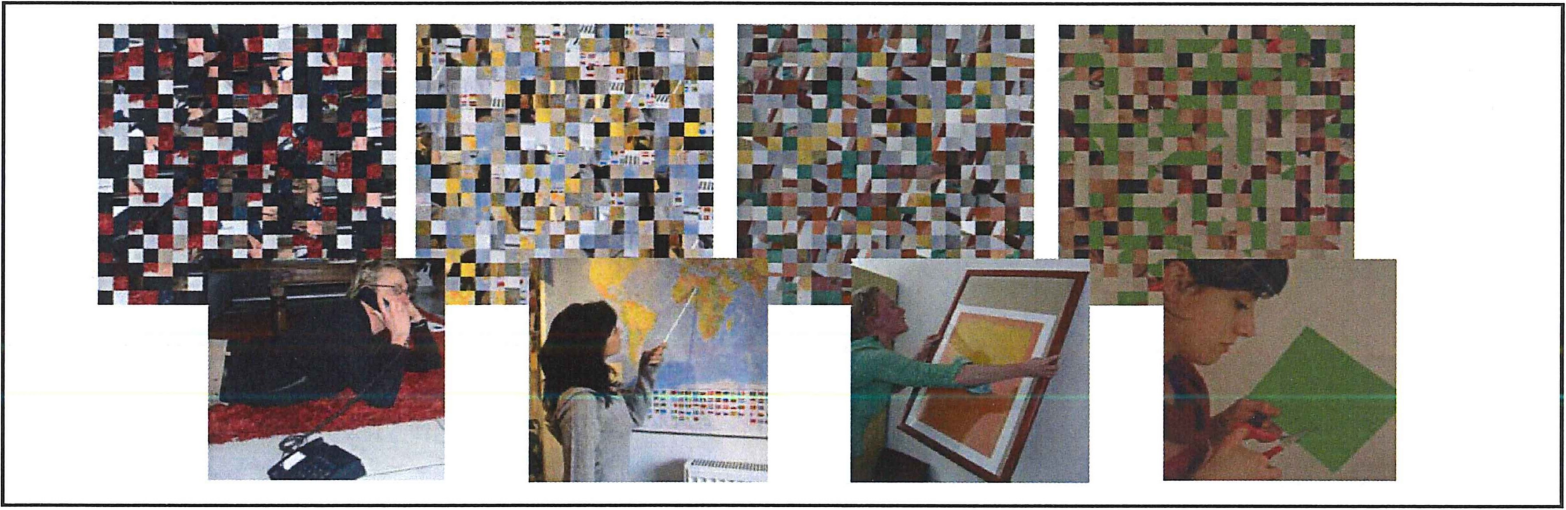
Examples of neutral primes used in Experiment 2.

The photo material was the same as in Experiment I, except for the fact that prime pictures in the Identical Condition were horizontally mirrored. In addition, a neutral prime type was created, by scrambling the original pictures (see Fig 2 for examples). With the *randblock toolbox* from *Matlab*, pictures were cut to pieces of 20 x 20 pixels, resulting in 400 pieces per picture, which were subsequently shuffled. Thirty pictures were randomly selected, ten from each prime type of Experiment I, and assigned to targets. Four different prime types (Identical, Same-Action, Neutral, Unrelated) were combined with each of the 30 targets. Each participant saw each action target four times (Presentation 1–4), once in each of the four prime types, resulting in 120 trials. In addition, 60 filler trials (30 with Neutral, 30 with Unrelated picture primes) were included to counterbalance prime-type frequency. Together with six practice trials, this resulted in 186 trials.

#### Design, apparatus & procedure

Participants were randomly assigned to one of four experimental lists, each with four blocks to implement target repetition (Presentation 1–4). The assignment of prime types to blocks and the randomization was the same as in Experiment 1. There were two factors: PRIME TYPE (Identical, Same-Action, Neutral, Unrelated) and TARGET REPETITION (Presentation 1–4). The same apparatus was used as in Experiment I. The primes were presented for 100 ms, followed by a mask. The experiment lasted approximately 25 minutes.

### Results

The same six items as in Experiment 1 were excluded. In case of significant main effects, *t*-tests for paired samples were calculated (*p* = .008 due to Bonferroni correction). Errors (6.1%) and extreme reaction times (less than 357 ms or above 1174 ms; 5.2% in total) were discarded from the data (see Table 2). The mean response time was 714 ms, 95% CI [690, 738] with Identical primes, 750 ms, 95% CI [730, 770] with Same-Action primes, 781 ms, 95% CI [759, 804] with Neutral primes, and 850 ms, 95% CI [830, 870] with Unrelated primes (see Table 2). The main effects for REPETITION, *F1*_(3, 87)_ = 104.54, *p* < .001, $\eta_{g}^{2}=.33$; *F2*_(3, 69)_ = 97.57, *p* < .001, $\eta_{g}^{2}=.38$, and PRIME TYPE, *F1*_(3, 87)_ = 119.39, *p* < .001, $\eta_{g}^{2}=.30$; *F2*_(3, 69)_ = 95.79, *p* < .001, $\eta_{g}^{2}=.34$, were significant. As in Experiment 1, latencies became shorter with repetition. All prime types yielded faster RTs than the Unrelated condition (see Table 3). Mean RT with Neutral primes was faster than with Unrelated primes (69 ms), but slower than with Identical (67 ms) and Same-Action (31 ms) primes. The interaction between TARGET REPETITION and PRIME TYPE reached significance (*F1*_(9, 261)_ = 2.04, *p* = .035, $\eta_{g}^{2}$ = .02*; F2*_(9,207)_ = 2.09, *p* = .032, $\eta_{g}^{2}=.03$). The interaction seems to be mainly due to the lack of a decrease in RT between subsequent presentations in two prime conditions (Unrelated, between presentations 2 and 3; Same-Action, between Presentations 3 and 4), where the other two show such decrease. Fig D in S2 File illustrates this interaction.

**Table 2:** Experiments 2 and 3, mean naming latencies in ms, standard deviations (between brackets) and percentage excluded responses as a function of Prime Type.

Experiment 2	*Prime Type*	RT (SD)	%
	*Identical*	714 (65)	10.6%
	*Same-Action*	750 (53)	10.2%
	*Neutral*	781 (60)	10.6%
	*Unrelated*	850 (54)	14.2%
Experiment 3			
	*Identical*	544 (60)	2.4%
	*Identical-Unrelated*	568 (61)	3.2%
	*Form-Related*	587 (65)	6.9%
	*Form-Unrelated*	577 (64)	5.5%

**Table 3:** Experiment 2, *t*-tests (over participants), all *p* < .005, *df* = 29.

Comparison	Mean Difference	*t*	*d*
unrelated—neutral	69	8.81	1.58
unrelated—same-action	100	12.20	2.19
unrelated—identical	136	18.30	3.29
same-action—identical	36	5.30	0.95
neutral—identical	67	11.42	2.05
neutral—same-action	32	3.73	0.67

#### Additional analysis

To test whether the horizontal mirroring in the identical prime condition had an influence on naming latencies, the data from Experiment 2 and Experiment 1B were entered into a repeated-measures ANOVA, with PRIME TYPE (Identical, Same-Action, Unrelated) as within-subject factor, and EXPERIMENT as between-subjects factor. The main effect of PRIME TYPE was significant, *F1*_(2, 116)_ = 344.11, *p* < .001, $\eta_{g}^{2}=.42$; *F2*_(2, 92)_ = 221.18, *p* < .001, $\eta_{g}^{2}=.58$, while EXPERIMENT was not (all *F* < 1). More importantly, there was no interaction between PRIME TYPE and EXPERIMENT, all *F* < 1. It is interesting to note that the priming effect induced by identical primes was large, whether mirrored (Experiment 2: 136 ms; Cohen’s d = 2.77) or not (Experiment 1B: 132 ms; Cohen’s d = 3.23).

### Discussion

The question addressed in Experiment 2 was whether effects by prime actions on the naming of subsequent action pictures are due to facilitation, interference, or both. The answer is both. Compared to a neutral prime that has the same basic visual properties than the other primes but does not provide any information about the spatial layout or meaning of actions, naming was faster in both related prime conditions, but slower in the unrelated condition. These results again provide clear evidence for the activation of conceptual information by briefly presented primes. But it qualifies our earlier results in that both interference and facilitation contribute to the priming effects. If this information is congruent with the target, faster naming is observed. If prime and target actions differ, naming is slowed. Given that visual similarity between same-action and unrelated prime-target pairs was controlled, we can exclude that effects were due to overall structural similarity only. It is interesting that the size of priming effects with 50 ms presentation (experiment 1c), calculated against the unrelated primes, is very similar to the size of priming effects in experiment 2, when contrasted to the neutral primes (57 / 67 ms for identical primes, 30/ 31 ms for same-action primes). This supports our interpretation advanced earlier that interference takes longer to develop than facilitation.

A second difference between Experiment 1 and 2 was the mirrored presentation of the primes in the identical condition. As predicted, effects were the same. This implies that the effect of identical primes is not due to purely visual similarity, facilitating early visual processing. Identical primes show the same action as the targets, with the same, orientation-independent layout. This explains their large facilitatory power on target processing. Clearly, same-action primes are also very effective, which again demonstrates that conceptual information is available early on [11, 12]. What we do not know is whether the observed effects are confined to the conceptual stratum or whether briefly presented and subsequently masked primes also activate lexical information. This question was explored in Experiment 3.

## Experiment 3: Picture-Word-Priming

With picture-word instead of picture-picture priming, we investigated whether briefly presented action scenes activate only semantic information or lexical (word-form) information as well. Our picture-picture paradigm with naming responses does not allow distinguishing between these options. We thus combined (most of) the prime stimuli from Experiments 1 and 2 with word targets that had to be named. The logic is as follows. When, as already shown in Experiment 1 and 2, the action prime activates semantic information, this should facilitate naming of the word denoting the same action (prime: picture of someone eating, target word “eat”), provided that word naming involves access to semantics [48], or that the semantic activation of the primes percolates to the word-form level. Data from the identical condition cannot distinguish these two options. If, however, the picture activates lexical information about the sound form associated with the concept (the word form *eat*), this word form should have an impact on related word forms, such as “beat”, presented as target for naming. Effects in this form-related condition would thus provide unequivocal evidence for activation of lexical-form information from masked action scenes.

### Method

#### Participants

Thirty participants (22 female, 19–43 years of age) took part in Experiment 3, all from the same pool as in Experiment 1 and 2, but none had participated in the earlier experiments. Participants were recruited between January and March 2010, provided oral informed consent, and received course credit or were paid for their participation.

#### Stimulus material

The primes consisted of 25 of the action pictures from Experiment 1. In addition, 15 similar photos from a previous experiment were used (also with more than 80% naming consistency). The main criterion for inclusion was whether a suitable form-related word target could be found for the picture name. Two sets of related targets were used; (1) the 40 German verbs denoting the action shown in the prime picture and (2) 40 German verbs that were phonologically related but semantically unrelated to the verb denoting the prime action. For phonological similarity, we chose rhyme overlap (e.g., the target word *tauchen*, to dive, combined with the action picture “rauchen”, to smoke). We considered rhyme overlap to better capture pure lexical effects, because onset overlap is confounded with positive effects on word naming due to sub-lexical (phonemic) similarity [44, 45]. The two related prime conditions, Identical and Form-Related, were each complemented by their own unrelated condition: Identical-Unrelated and Form-Unrelated. These were created by reassigning the verb targets to semantically or phonologically unrelated pictures from the prime set (e.g., picture “rauchen”, to smoke, with target word *schenken*, to donate). Note that this way, the same action primes and word targets served as related and unrelated primes. Whereas target words were presented twice (with related and unrelated picture primes), the same prime pictures were used in all conditions, and thus repeated four times.

#### Design, apparatus, & procedure

Four experimental lists with four blocks each were created, each with one of the four prime actions, combined with one of the two targets. These blocks were randomized separately for each list and implemented prime repetition. Together with six practice trials, this resulted in 166 action-word pairs. Each participant saw each action prime four times, once in each condition. There were three factors in the experiment: PRIME TYPE (Identical, Form-related), RELATEDNESS (Related, Unrelated) and PRIME REPETITION (Presentation 1–4).

The same apparatus was used as in Experiment 1 and 2. Action primes were presented for 100 ms, as in Experiment 2. The procedure differed in that words, not pictures, were presented as targets for naming, for a maximal duration of 1500 ms, disappearing upon speech onset. The task was to read the word aloud as quickly and accurately as possible. The experiment lasted about 15 minutes.

### Results

Speech-onset latencies were submitted ANOVAs with PRIME TYPE (Identical, Form), RELATEDNESS (Related, Unrelated) and PRIME REPETITION (Presentation 1–4) as factors. PRIME TYPE was a within factor in the F1-analysis, but a between factor in the F2-analysis. T-tests for paired samples assessed the significance of effects. The error rate was 1.4%, and trials with extreme reaction times (less than 292 ms or above 842 ms) amounted to 3.0%. These were removed from the data set for the latency analysis, and so were all data for the target “longieren” (to lunge), because there were more than 20% errors. The data are shown in Table 2.

Mean naming latency was 545 ms, 95% CI [522, 567] with Identical primes, 568 ms, 95% CI [545, 590] in the Identical-Unrelated condition, 587 ms, 95% CI [562, 611], with Form primes, and 577 ms, 95% CI [553, 600] in the Form-Unrelated condition (see Table 2). The main effect for PRIME TYPE was significant (*F1*_(1, 29)_ = 107.43, p < .001, $\eta_{g}^{2}$ = .03; *F2*_(1, 77)_ = 14.57, *p* < .001, $\eta_{g}^{2}=.06$) as were the main effects of RELATEDNESS (*F1*_(1, 29)_ = 10.95, *p* = .003, $\eta_{g}^{2}<.01$; *F2*_(1, 77)_ = 8.44, *p* = .005, $\eta_{g}^{2}<.01$) and PRIME REPETITION (*F1*_(3, 87)_ = 2.79, *p* = .045, $\eta_{g}^{2}=.01$; *F2*_(3,231)_ = 5.01,p = .002, $\eta_{g}^{2}=.02$, indicating that responses became faster over blocks. Importantly, the interaction of PRIME TYPE and RELATEDNESS was also significant (*F1*_(3, 87)_ = 73.15, *p* < .001, $\eta_{g}^{2}=.01$; *F2*_(1, 77)_ = 47.01, *p* < .001, $\eta_{g}^{2}=.03$). Both effects, facilitation by pictures whose name was identical to the word target, and interference by pictures whose name was form-related to the target word, were significant. Responses to identical related targets were faster 24 ms than responses to their matched unrelated targets (*t1*_(29)_ = -8.63, *p* < .001, *d* = -1.55; *t2*_(39)_ = -7.40, *p* < .001, *d* = -1.16)). In contrast, responses to form-related targets were 10 ms slower than responses to form-unrelated targets (*t1*_(29)_ = 3.67, *p* < .001, *d* = .66; *t2*_(38)_ = 2.66, *p* = .011, *d* = .42). None of the other interactions was significant (*F* < 1); see also Fig E in S2 File.

### Discussion

Experiment 3 was designed to investigate whether briefly presented action photos activate lexical information–the name for the action–in addition to semantic information. We used action pictures as primes, and action verbs as targets. The relation between action primes and word targets was either name identity, or word-form similarity. The identity condition revealed 24 ms facilitation that could be interpreted as an effect at the semantic level, where the word accesses its meaning, which is primed by the picture. An alternative interpretation would be that the action picture activates its name, which happens to be the target for word naming. Direct evidence for lexical activation by action photos comes from the form condition. The overall effect, 10 ms interference due to related as compared to unrelated action primes, was significant. This finding supports the idea that pictures activate their phonological information. Others have shown that pictures activate their words forms when fully available for inspection, even if they do not have to be named [cf. 34, 36, 38, 39]. What is new is the fact that no more than 100 ms of picture presentation, preventing effective eye-movements and thus extensive visual exploration, is needed for lexical activation. Whereas facilitation due to form overlap can be interpreted as arising from sub-lexical levels [45], interference is a lexical effect, due to response competition between form-similar words. The mere presence of form inhibition is evidence for lexical involvement. Thus, action pictures activate their word forms, although they are only briefly flashed, never have to be named in this particular experiment, and, if used to initiate the response, would induce an incorrect response on 75% of the trials.

## General discussion and conclusions

We assessed the availability of conceptual and lexical information from briefly flashed natural action pictures that served as primes to pictures or words that had to be named. Prime pictures were masked, to constrain visual uptake to the exposure duration. The exposure duration of primes was varied and could be as short as 50 milliseconds. Action primes and target pictures had the following types of relation: they were (1) identical (albeit mirrored, in Experiment 2), (2) showed the same action but with different layout, actors and objects, or (3) or unrelated. The rationale for using briefly presented action stimuli as task-irrelevant primes was to assess their automatic processing, without explicit, task-induced attention to these stimuli. If reactions to flashed stimuli are measured, in tasks such as categorization, or patient detection, the stimuli are highly relevant and may well be processed according to the task demands that induce strategic effects. As primes, the action scenes are task-irrelevant and could have been ignored, which obviously is not possible when stimuli have an abrupt onset [49]. Note that the exposure durations were too short for the scanning, by eye movements, of elements in the scene. In fact, even with 150 ms presentation time, no more than a first saccade could have been launched, before the picture was masked.

In Experiment 1, we obtained robust facilitation of picture naming from identical and same-action primes, relative to unrelated ones, even with the shortest prime presentation. Experiment 2, with neutral primes, showed that effects were two-sided, with facilitation by related primes, and interference by unrelated ones. Both interference and facilitation provide clear evidence for the activation of conceptual-semantic information by briefly presented action primes. Even 50 ms of exposure sufficed to access the action prime’s concept. A comparison of the data from Experiments 1 and 2 further showed that mirrored action scenes generated the same results as primes and targets with the same orientation. This fits well with the suggestion made for objects by Biederman and Gerhardstein [47] that recognition is normally viewpoint-invariant, unless exact orientation is task-relevant.

Note that we were not interested in whether primes reached consciousness for any of the presentation durations. We merely wished to constrain information uptake from the outside event, the prime, to a period that prevents visual inspection of scene details by means of eye movements. Internal processing is not prevented by our type of visual mask, and the fact that the prime pictures activated their semantic (and phonological) information shows that such processing indeed took place. There is evidence for semantic activation of truly subliminal pictures in semantic decision tasks [13], and it is known that in such decision tasks with few alternative responses, effects are the same, whether primes remain subliminal or are perceived consciously [50].

The data from Experiments 1 and 2 corroborate and clearly extend findings by others. First, the information provided by briefly flashed, natural scenes seems far more detailed than often assumed. Within one glance and without focused attention to individual scene details, the actual action performed in the scene is identified to such an extent that it primes the processing of subsequently presented action scenes, and their names. Note that in only a few pictures, they key action information was prominent and near to the fixation point (e.g., drilling, with a large drill). Sometimes, the relevant information was peripheral (telephoning), or spread out over the picture (fishing). Quite often a small and/or peripheral object (sewing, with a small needle, whistling) was critical for action naming. In all, it is certainly not the case that the verb specifying the action could be gathered from one centrally present object or action. In this light, such a fast uptake of action information was not expected on the basis of earlier data [1, 19]. Previous results demonstrated that upon brief exposure, complex pictures could be processed to such an extent that categorization or coherence judgments were possible [4, 23]. Note, however, that Glanemann [26] already showed that, after a briefly presented and subsequently masked action scene, internal visual representations can be detailed enough to generate correct agent-patient relations; see also [28]. Fei-Fei et al. [17] showed that information about objects can be accessed from peripheral vision, and Van den Bussche et al. [13] reported priming by masked picture primes when these triggered the same response (semantic category decision) as the target word.

We extend these findings to show that semantic and conceptual information about actual actions is available to the extent that the naming of a subsequent action is facilitated. Thus, we confirm conclusions by Potter and colleagues [11, 12] that brief visual presentations of highly complex scenes suffice to access very detailed information. What we show here that this happens rapidly, with little time between the action prime and the response to the target. Using less natural tasks, such as forced choice saccadic tasks, seems to generate different results [10]. What our data show is that there is more information available from briefly presented pictures than can be gathered by direct answers to questions asked about the pictures–and this we demonstrate by means of priming.

Next, the data from Experiment 3, with words instead of pictures as targets, revealed shorter word-naming latencies when the action specified by the prime and the verb target mapped onto the same concept. This convergence at the semantic level seems a parsimonious interpretation, assuming semantic access from written words that merely have to be read out loud–a valid assumption, given what reading is good for. Alternatively, the convergence might be at the lexical, word-form level, provided that the prime scene activates its lexical information: the name for the action. Evidence that this is the case comes from the condition with action primes and verb targets that were merely phonologically related. The results from experiment 3 revealed interference when the name of the prime action and the written target word were related in form (but not in meaning). This can only come about because the prime action activated its word form. As often the case in word naming, the effect was numerically small [51], but its significance strongly indicates that the action pictures briefly shown as primes activate their lexical information.

The finding that phonological information is available from pictures that are task-irrelevant and do not have to be named corroborates results by others [34, 38, 39], but note that the irrelevant pictures were presented for much longer durations in these studies. The data also fit with the findings of Levelt et al. [40], who observed interference effects from picture names on phonologically related words in a lexical decision task. In this study, however, all pictures had to be named, even the ones preceding a word target. Thus, what their data show is that the phonological encoding of the picture was well underway. Finally, it should be kept in mind that pictures of simple objects were presented in those earlier studies, not complex action scenes. Thus, it is surprising and exciting that we obtained evidence for word-form activation in the third experiment.

In our study, participants were in speech production mode, even though the prime scenes never had to be named. Some of the prime names were used in target naming, but effects are not confined to these situations. The interference by unrelated scene primes (Experiment 2) and the effects of phonologically related primes (Experiment 3) are cases in point. If participants had initiated naming on the basis of the primes, counting filler trials, they would have initiated a wrong response in 50% (Experiment 1) to 75% (Experiment 3) of the cases. In fact, there were hardly any prime names among the wrong responses to targets in all experiments. Moreover, if naming had been initiated by the primes, we would have expected similar amounts of priming in identical and same-action conditions, which is not what we found.

Taken together, by using a task that is highly natural, well investigated and rather well understood, that is, naming in the context of primes, we addressed a long-standing and still open question: how deeply are briefly presented, complex pictures processed? The results from three experiments together demonstrate that they are indeed processed to a rather deep level, even to modality-independent representations. With regard to naming and describing complex scenes, Griffin and Bock [24] argued–in line with Wilhelm Wundt’s theory on sentence production–that an apprehension phase is being followed by a formulation phase. In their study the apprehension phase was characterized by a series of eye movements before formulation started. It was argued that the first eye movement for the description of an action scene is directed towards a scene region that contains information about the action, later to be described by a verb [52]. Later studies casted doubt on the assumption that (covert) attention shifts are necessary to describe actions and actors [23, 53, 54]. In the current study, we show that an apprehension phase for retrieval of a verb can be extremely short and is not necessarily marked by attention shifts. It appears that action scenes activate their word forms (here: verbs), even when scenes do not have to be named [34, 39]. We do not know if this would still be the case when participants are not in “talking mode”, as in the study of Chaban and Marian [39], but if they are, a brief glimpse suffices to proceed through all levels of word production [31] except for articulation.

### Limitations

We acknowledge that the task used to assess the availability of information gathered from briefly flashed pictures can have a large impact on the results. First, providing a response to briefly flashed pictures seems to be different from using such stimuli as primes. Second, the retrieval of action information may be different when the action verbs are used in sentential action descriptions [24] instead of as a single verbal response, as was the case in our study. Next, we used masking to avoid further retinal input for internal processing. This raises the question how such short presentations can lead to deep processing. First, it should be kept in mind that the time for internal processing clearly extends the time of stimulus presentation. Moore and Wolfe [55] used the carwash metaphor to describe that a single processing pipeline suffices to work on different stimuli at the same time, by passing them from one step to the next. For example, several pictures presented in sequence can be processed at the same time (at different levels), and in the end all have accessed their conceptual information [11]. For the description of complex scenes, when the processing pipeline is hooked up with language production, scenes even activate their phonological information.

In conclusion: At the interface between visual perception and speech production, we demonstrated that a briefly flashed, visually complex action scene already provides enough information to influence the naming of an immediately following action picture, or even action word. Thus, when in speaking mode, visual information uptake from limited input is effective and thorough enough to provide lexical information. We strongly believe that the priming paradigm is worth its while to address the availability of semantic and lexical information in scene perception, because it does not require responses to the stimulus of interest. It can easily be combined with tasks other than naming, and is thus ideally suited for further explorations of semantic and lexical access after brief exposure to complex visual scenes.

### Appendix: List of actions in Experiments 1 and 2

**Table:** 

German verb	English translation
abhören	to auscultate
angeln	to fish
aufhängen	to hang up
bohren	to drill
bügeln	to iron
essen	to eat
flüstern	to whisper
fotografieren	to photograph
frieren	to freeze
gießen	to water
kochen	to cook
lachen	to laugh
lesen	to read
malen	to paint
nähen	to sew
radfahren	to cycle
rasieren	to shave
schälen	to peel
schenken	to make a gift
schlafen	to sleep
schneiden	to cut
schreiben	to write
singen	to sing
surfen	to surf
tanzen	to dance
telefonieren	to phone
treten	to kick
trinken	to drink
winken	to wave
zeigen	to point

## Supporting information

S1 File Linear mixed effect analyses for all experiments. (DOCX)

S2 File Figures A—E illustrating interactions for all experiments.
(DOCX)
