# Supplementary material for: Seeing for speaking: Semantic and lexical information provided by briefly presented, naturalistic action scenes
Source: PLoS One. 2018 Apr 13;13(4):e0194762. doi: 10.1371/journal.pone.0194762 (PMC5898714; doi:10.1371/journal.pone.0194762)
Supplement: S1 File — (DOCX) [file pone.0194762.s001.docx]

# **Linear mixed effects analyses for all experiments**

In addition to ANOVAs, we calculated LME (Baayen, Davidson, & Bates, 2008), trying achieve a parsimonious random effect structure justified by the data (Barr, Levy, Scheepers, & Tily, 2013; Bates, Kliegl, Vasishth, & Baayen, 2015; Kuznetsova, Brockhoff, & Christensen, 2016). Data were analyzed using R 3.4.3 (R Core Team, 2017). We applied a-priori trimming, excluding erroneous answers and time-outs as defined in the ANOVA analyses. We used a treatment contrast (the default in R) for the factor Prime Type, with the unrelated prime condition serving as reference for priming effects of related prime conditions. This contrast resembles the post-hoc t-tests run after the ANOVAs to check for priming effects. A successive difference contrast (e.g., 1. Presentation vs. 2. Presentation, 2. Presentation vs. 3. Presentation ) was used for the factor Repetition.

The use of an instrument in the action served as a predictor, with 1 coding instrument use and 0 coding no instrument use (Instrument, not centered). We included the following (centered) continuous predictors: normalized lemma target frequency (ctfreq), normalized lemma prime frequency (cpfreq; Heister, Würzner, Bubenzer, Pohl, Hanneforth et al. 2011), trial number (ctrial) target length in number of letters (ctlength), prime length (cplength) in number of letters. These served as control predictors.

The models were fit by restricted maximum likelihood (REML). To assess the relative goodness of fit, lmer offers the AIC (Akaike information criterion which decreases with goodness of fit), the BIC (Bayesian Information Criterion which also decreases with goodness of fit), LogLik (log-likelihood, which increases with goodness of fit) and, for model comparisons, the χ²-distributed likelihood ratio along with its p-value. The AIC (that corrects the LogLik for the number of estimates) and the BIC (that corrects the LogLik for the number of observations) were used to protect against overfitting in the model selection process.

We started with the most complex model (random slopes for participants and items, and random intercepts). When a model did not converge, it was simplified by replacing the interactions of Repetition and Prime Type in the random slope terms by main effects. Then main effects were deleted from the participants’ random slope term (Prime Type first, then Repetition). Finally, the remaining random slopes were simplified by deleting the main effects from the item term if the simplification did not result in significant decrease in fit indicated by significant χ²-test (Matuschek, Kliegl, Vasishth, Baayen, & Bates, 2017).

## **Experiment 1a (150 ms prime duration)**

The best fitting model differed from the random intercept model (χ²(5) = 20.58, *p* < .001) and all other models. The main effects of Repetition (*F*(2, 106.56) = 26.42, *p* < .001) and Prime Type (*F*(2, 1473.53) = 147.32, *p* < .001) were significant in an ANOVA Type III using a Satterthwaite approximation for the df (Kuznetsova, Brockhoff, & Bojesen Christensen, 2017). Furthermore, the interaction of Repetition and Prime Type was significant (*F*(4, 1467.81) = 2.66, *p* = .032; see Table 1a for a summary of the contrasts). RT in the identical and the same-action condition differed significantly from the RT in unrelated condition (*t*(1456.7) = -17.07, *p* < . 001; *t*(1475.3) = -10.36, *p* < .001). RT decreased from presentation to presentation. All but one combination of Repetition and Prime Type could be explained by the additive main effects of Repetition and Prime Type only. The exception is the second presentation of an identical prime, which needs an additional parameter (the interaction of Repetition and Prime Type, *t*(1462.4) = -2.975, *p* = .003) to be estimated. This interaction was due to larger decrease from the first to second presentation in the identical condition than in the unrelated condition (see also Figure 1). In sum, participants named action faster from presentation to presentation, and naming was faster with identical and same-action primes relative to unrelated primes. The correlation between the predicted value of the best fitting model rt ~ Repetition * Prime Type + ctfreq + cpfreq + ctrial + ctlength + cplength + instrument + (1 + Repetition | vp) + (1 | target) and observed RT was *r* = .678.

**Table 1a: Output of the best-fitting linear mixed-effects model on the RT for Experiment 1a**

**(Model: rt ~ Repetition * Prime Type + ctfreq + cpfreq + ctrial + ctlength + cplength + instrument + (1 + Repetition | vp) + (1 | target))**

| Fixed | Estimate | Std. Error | df | t value | Pr(>\|t\|) |
| --- | --- | --- | --- | --- | --- |
| (Intercept) | 744.54 | 25.18 | 45.1 | 29.57 | < .001 |
| 1. vs. 2. presentation | -98.43 | 22.18 | 211.4 | -4.437 | < .001 |
| 2. vs. 3. presentation | -37.91 | 21.33 | 760.4 | -1.777 | .077 |
| unrelated vs. same action | -95.96 | 9.26 | 1475.3 | -10.36 | < .001 |
| unrelated vs. identical | -158.21 | 9.27 | 1465.7 | -17.07 | < .001 |
| ctfreq | 0.02 | 0.10 | 25.7 | 0.154 | .878 |
| cpfreq | -0.01 | 0.05 | 1467.7 | -0.075 | .940 |
| ctrial | 0.29 | 0.33 | 1483.5 | 0.879 | .380 |
| ctlength | 0.93 | 5.12 | 25.4 | 0.181 | .857 |
| cplength | -4.71 | 2.78 | 1428.1 | -1.697 | .089 |
| instrument | 6.90 | 22.67 | 20.0 | 0.304 | .764 |
| 2. presentation : same action | -20.33 | 22.60 | 1465.9 | -0.899 | .369 |
| 3. presentation : same action | -7.51 | 22.19 | 1465.4 | -0.339 | .735 |
| 2. presentation : identical action | -67.16 | 22.58 | 1462.4 | -2.975 | .003 |
| 3. presentation : identical action | -12.17 | 22.31 | 1462.9 | 0.545 | .586 |

## **Experiment 1b (100 ms prime duration)**

The same predictors were included as in Experiment 1a, but a different random effect structure emerged. The best fitting model included Repetition as random slope effect for participants and items. The correlation of fitted and observed RT was *r* = .72. It outperformed the most simple model with random intercepts only (χ²(10) = 76.43, *p* < .001) as well as the remaining models. An ANOVA Type III showed that the main effect of Repetition and Prime Type (*F*(2, 51.74) = 21.37, *p* < .001; *F*(2, 1814.39) = 221.20, *p* < .001) were significant, while the interaction was not (*F*(4, 1807.82) = 0.94, *p* = .441). The contrast analysis showed the identical and same-action priming effects to be significant. Two predictors were significant: Prime Length (*F*(1, 1689.03) = 8.77, *p* = .003) and Target Frequency (*F*(1, 28.29) = 4.84, *p* = . 036; see Table 1b for the output of the best-fit linear mixed-effects model). The significant effect or Prime Length indicates that RT decreased with increasing prime length (of the name of the prime picture, in number of letters). Unexpectedly, more frequent target picture names resulted in slower RTs.

**Table 1b: Output of the best-fitting linear mixed-effects model on the RT for Experiment 1b (Model: rt ~ Repetition * Prime Type + ctfreq + cpfreq + ctrial + ctlength + cplength + instrument + (1 + Repetition | vp) + (1 + Repetition | target))**

| Fixed | Estimate | Std. Error | df | t value | Pr(>\|t\|) |
| --- | --- | --- | --- | --- | --- |
| (Intercept) | 825.05 | 19.96 | 56.8 | 41.34 | < .001 |
| 1. vs. 2. presentation | -95.42 | 18.86 | 114.6 | -5.06 | < .001 |
| 2. vs. 3. presentation | -32.31 | 16.95 | 181.1 | -1.91 | < .06 |
| unrelated vs. same action | -90.51 | 6.47 | 1821.1 | -13.98 | < .001 |
| unrelated vs. identical | -133.70 | 6.45 | 1811.8 | -20.73 | < .001 |
| ctfreq | 0.171 | 0.08 | 28.3 | 2.20 | .036 |
| cpfreq | -0.07 | 0.04 | 1813.3 | -1.96 | .050 |
| ctrial | 0.41 | 0.242 | 1806.9 | 1.70 | .089 |
| ctlength | 1.56 | 3.96 | 27.9 | 0.39 | .697 |
| cplength | -5.80 | 1.97 | 1690.8 | -2.96 | .003 |
| instrument | 22.83 | 17.94 | 23.5 | 1.27 | .216 |
| 2. presentation : same action | -4.34 | 15.74 | 1810.9 | -0.28 | .783 |
| 3. presentation : same action | 7.36 | 15.58 | 1808.2 | 0.47 | .636 |
| 2. presentation : identical action | -26.98 | 15.71 | 1811.8 | -1.72 | .086 |
| 3. presentation : identical action | -11.17 | 15.50 | 1808.0 | 0.72 | .471 |

## **Experiment 1c (50 ms prime duration)**

The model which included Repetition as random slope outperformed the random-intercept only model (χ²(5) = 57.70, *p* < .001) and all other models tested. The linear mixed-effect model analysis showed significant effects for Repetition (*F*(2, 39.47) = 26.11, *p* < .001), Prime Type (*F*(2, 1831.96) = 35.29, *p* < .001) in an ANOVA Type III (see Table 1c for a summary of the contrasts). The use of an instrument in the action was also significant (*F*(1, 20.05) = 6.35, *p* = .020) showing that voice key latencies for actions with an instrument were slower than latencies for actions without an instrument. Keep in mind that this comparison involves different items that were not matched, which might have contributed to the effect irrespective of whether the action involved an instrument or not. The best fitting model had a random intercept for participants and a random intercept and slope for targets: rt ~ Repetition * Prime Type + ctfreq + cpfreq + ctrial + ctlength + cplength + instrument + (1 | vp) + (1 + Repetition | target). The correlation of the fitted and observed RT was r = .71.

**Table 1c: Output of the best-fitting linear mixed-effects model on the RT for Experiment 1c (Model: rt ~ Repetition * Prime Type + ctfreq + cpfreq + ctrial + ctlength + cplength + instrument + (1 | vp) + (1 + Repetition | target))**

| Fixed Effects | Estimate | Std. Error | df | t value | Pr(>\|t\|) |  |
| --- | --- | --- | --- | --- | --- | --- |
| (Intercept) | 753.52 | 22.43 | 59.4 | 33.58 | < .001 |  |
| 1. vs. 2. presentation | -87.32 | 17.79 | 122.1 | -4.91 | < .001 |  |
| 2. vs. 3. presentation | -50.79 | 15.04 | 300.9 | -3.38 | < .001 |  |
| unrelated vs. same action | -27.65 | 6.53 | 1833.8 | -4.23 | < .001 |  |
| unrelated vs. identical | -54.89 | 6.53 | 1836.5 | -8.40 | < .001 |  |
| ctfreq | 0.07 | 0.078 | 25.6 | 0.90 | .375 |  |
| cpfreq | 0.01 | 0.036 | 1845.6 | 0.17 | .867 |  |
| ctrial | 0.39 | 0.24 | 1345.0 | 1.61 | .108 |  |
| ctlength | 1.51 | 4.02 | 25.2 | 0.38 | .709 |  |
| cplength | -0.31 | 1.92 | 1797.7 | -0.16 | .869 |  |
| instrument | 44.96 | 17.84 | 20.1 | 2.52 | .020 |  |
| 2. presentation : same action | -30.76 | 15.93 | 1830.9 | -1.93 | .053 |  |
| 3. presentation : same action | 11.48 | 15.66 | 1831.5 | 0.73 | .463 |  |
| 2. presentation : identical action | -28.82 | 15.91 | 1832.2 | -1.81 | .070 |  |
| 3. presentation : identical action | -9.59 | 15.64 | 1833.5 | -0.61 | .539 |  |

The contrast analyses showed both priming effects to be significant unrelated vs. identical condition (*t*(1836.5) = -8.40 *p* < .001); unrelated vs. same-action condition (*t*(1833.8) = -4.23, *p* < .001)

# **Experiment 2: Picture-Picture-Priming with neutral primes**

The effects of Prime Type (*F*(3, 22.42) = 95.40, *p* < .001) and Repetition (*F*(3, 37.76) = 24.67, *p* < .001) were significant, with significant differences between the unrelated prime condition and all others (identical action, same action, neutral). Two predictors reached significance: Target frequency (*F*(1, 23.70) = 14.44, *p* < .001) and Instrument (*F*(1, 21.25) = 12.35, *p* = 0.002). As before, RTs increased with increasing target-word frequency, and the use of an instrument in the action slowed reaction times. As in the ANOVAs, the interaction of Repetition and Prime type was significant (*F*(9, 2384.23) = 3.51, *p* < .001). The selected model outperformed the random intercept model (χ²(27) = 85.71, *p* < .001) and other models. The correlation of the fitted and observed RT was *r* = .71 (see Table 2 for a summary of the contrasts and the model structure).

**Table 2: Output of the best-fitting linear mixed-effects model on the RT for the picture-picture experiment 2 (Model: rt ~ Repetition * Prime Type + ctfreq + cpfreq + ctrial + ctlength + cplength + instrument + (1 | vp) + (1 + Repetition + Prime Type | target)**

| Fixed effects | Estimate | Std. Error | df | t value | Pr(>\|t\|) |
| --- | --- | --- | --- | --- | --- |
| (Intercept) | 822.45 | 15.61 | 52.5 | 52.70 | < .001 |
| 1. vs. 2. presentation | -103.53 | 16.27 | 137.7 | -6.36 | < .001 |
| 2. vs. 3. presentation | -16.51 | 14.66 | 439.9 | -1.12 | .261 |
| 3. vs. 4. presentation | -40.29 | 13.94 | 1006.7 | -2.89 | .004 |
| identical | -134.67 | 8.63 | 22.2 | -15.61 | < .001 |
| same action | -98.18 | 7.44 | 21.0 | -13.20 | < .001 |
| neutral | -69.00 | 7.97 | 20.9 | -8.65 | < .001 |
| ctfreq | 0.23 | 0.06 | 23.7 | 3.80 | < .001 |
| cpfreq | -0.04 | 0.03 | 37.4 | -1.20 | .239 |
| ctrial | 0.32 | 0.17 | 1127.2 | 1.86 | .063 |
| ctlength | 2.86 | 3.05 | 22.0 | 0.94 | .360 |
| cplength | -1.95 | 1.95 | 35.7 | -1.00 | .324 |
| instrument | 47.42 | 13.49 | 21.2 | 3.51 | .002 |
| 2. presentation : identical | -27.11 | 16.73 | 2381.2 | -1.62 | .105 |
| 3. presentation : identical | -35.41 | 16.44 | 2377.2 | -2.15 | < .031 |
| 4. presentation : identical | -0.98 | 16.35 | 2382.0 | -0.06 | .952 |
| 2. presentation : same action | 14.17 | 16.83 | 2388.6 | 0.84 | .400 |
| 3. presentation : same action | -58.24 | 16.41 | 2387.8 | -3.55 | < .001 |
| 4. presentation : same action | -22.13 | 16.33 | 2380.1 | 1.35 | .176 |
| 2. presentation : neutral | 8.98 | 16.84 | 2381.3 | 0.53 | .594 |
| 3. presentation : neutral | -42.89 | 16.43 | 2383.8 | -2.61 | .009 |
| 4. presentation : neutral | 23.51 | 16.36 | 2382.3 | 1.44 | .151 |

# **Experiment 3: Picture-Word-Priming**

We conducted the analysis as reported above with the following changes. Instrument use was not included as control predictor because the targets were words, not pictures. Furthermore, target and prime frequency were scaled in addition to being centered, because they were on a very different scale than the other predictors. This problem arose because we added some new high-frequency items that had not been used in experiments 1a,b,c and 2.

The selected model outperformed the random intercept model (χ²(9) = 312.61, *p* < .001) and other models. We used the default contrast for Relatedness (related, unrelated) and Prime Type (identical, form-related). Repetition was again investigated with a contrast of successive differences. The analysis revealed a significant effect of Prime Type (*F*(1, 73.4) = 23.44, *p* < .001). The effect of Relatedness (*F*(1, 4257) = 8.90. *p* = .003) was also significant, showing differences between both related and unrelated prime conditions. Most importantly, the interaction of Prime Type and Relatedness was significant (*F*(1, 4259.4) = 67.10, *p* < .001). Whereas reactions to target words were faster when preceded by identical pictures (*t*(4311.7) = -8.10, *p* < .001), form-related procures slowed word naming (*t*(4316.3) = 3.60, *p* = .003). The main effect of Repetition was not significant (*F*(3, 37.9 ) = 0.71, *p* = .549), neither were the interactions of Repetition and Prime Type (*F*(3, 4256.1) = 0.244, *p* = .865), Repetition and Relatedness (*F*(3, 4256.6) = 0.90, *p* = .440) nor the three-way interaction (*F*(3, 4257.4) = 0.73, *p* = .533). One predictor, target length (in number of letters) was significant (*F*(1, 80.1) = 7.44 *p* = .007). Not surprisingly, reaction times increased with increasing target length (see Table 3 for a summary of the best-fit linear mixed-effects model). The correlation of the fitted and observed RT was *r* = .73.

**Table 3: Output of the best-fitting linear mixed-effects model on the RT for the picture-word experiment (Model: rt ~ Repetition * pt * related + ctfreq + cpfreq + ctrial + ctlength + cplength + (1 + Repetition | vp) + (1 | target))**

| Fixed Effects | Estimate | Std. Error | df | t value | Pr(>\|t\|) |
| --- | --- | --- | --- | --- | --- |
| (Intercept) | 589.81 | 12.13 | 39 | 48.63 | < .001 |
| 1. vs. 2. presentation | -8.587 | 9.00 | 104 | -0.95 | .342 |
| 2. vs. 3. presentation | 2.423 | 8.47 | 136 | 0.29 | .775 |
| 3. vs. 4. presentation | -9.21 | 7.84 | 234 | -1.17 | .242 |
| Prime Type | -46.73 | 6.53 | 90 | -7.15 | < .001 |
| Relatedness | -10.60 | 2.95 | 4260 | -3.60 | < .001 |
| ctfreq | -5.93 | 3.13 | 73 | -1.89 | .062 |
| cpfreq | -0.09 | 1.33 | 3382 | -0.06 | .949 |
| ctrial | -0.03 | 0.09 | 4297 | -0.40 | .687 |
| cplength | 7.18 | 2.61 | 80 | 2.75 | .007 |
| ctlength | -0.95 | 1.18 | 3338 | -0.81 | .419 |
| 2. presentation : Prime Type | -0.30 | 8.18 | 4256 | -0.04 | .971 |
| 3. presentation : Prime Type | -3.24 | 8.10 | 4254 | -0.40 | .689 |
| 4. presentation : Prime Type | 11.39 | 8.12 | 4255 | 1.40 | .161 |
| 2. presentation : Relatedness | -0.09 | 8.39 | 4258 | -0.01 | .992 |
| 3. presentation : Relatedness | -8.36 | 8.26 | 4257 | -1.01 | .312 |
| 4. presentation : Relatedness | 4.40 | 8.264 | 4256 | 0.53 | .594 |
| Prime Type : Relatedness | 33.35 | 4.07 | 4257 | 8.19 | < .001 |
| 2. presentation : Prime Type : Relatedness | -2.48 | 11.59 | 4256 | -0.21 | .831 |
| 3. presentation : Prime Type : Relatedness | 10.03 | 11.45 | 4255 | 0.88 | .381 |
| 4. presentation : Prime Type : Relatedness | -16.78 | 11.46 | 4254 | -1.47 | .143 |

# **Discussion**

All LME analyses show the same pattern of results as the traditional analyses of *F1* and *F2*: main effects of Repetition and Prime Type, and significant priming effects in all experiments and prime conditions. Only one minor difference between the two types of statistical model was observed: a significant (but not very interesting) interaction of Repetition and Prime Type in Experiment 1a (see Figure 1). Control predictors that were not included in the ANOVAs showed some impact: word naming was slowed as a function of word length in Experiment 3 – a well-established effect in word recognition. Next, picture-naming latencies were faster when the names of the primes were long, but the frequency of the target-picture names slowed naming. It is important to consider that different items (targets or primes) are compared here, which may well differ on many other aspects than the one represented by the predictor. This is not the case for effects of the experimental variables (Prime Type, Repetition, Relatedness), for which the same targets are considered.

**References**

Baayen, R. H., Davidson, D. J., & Bates, D. M. (2008). Mixed-effects modeling with crossed random effects for subjects and items. *Journal of Memory and Language*, *59*(4), 390–412. doi:10.1016/j.jml.2007.12.005

Barr, D. J., Levy, R., Scheepers, C., & Tily, H. J. (2013). Random effects structure for confirmatory hypothesis testing: Keep it maximal. *Journal of Memory and Language*, *68*(3), 255–278. doi:10.1016/j.jml.2012.11.001

Bates, D., Kliegl, R., Vasishth, S. & Baayen, H. (2015). Parsimonious mixed models. *arXiv preprint arXiv:1506.04967*.

Heister, J., Würzner, K.-M., Bubenzer, J., Pohl, E., Hanneforth, T., Geyken, A. & Kliegl, R. (2011) dlexDB - eine lexikalische Datenbank für die psychologische und linguistische Forschung. *Psychologische Rundschau*, 62(1):10-20.

Kuznetsova, A., Brockhoff, P. B. & Bojesen Christensen, R., H. (2017). lmerTest: Tests in Linear Mixed Effects Models. R package version 2.0-36. <https://CRAN.R-project.org/package=lmerTest>

Matuschek, H., Kliegl, R., Vasishth, S., Baayen, H., & Bates, D. (2017). Balancing Type I error and power in linear mixed models. *Journal of Memory and Language*, *94*, 305–315. <https://doi.org/10.1016/j.jml.2017.01.001>

R Core Team (2017). R: A language and environment for statistical computing. R Foundation for Statistical Computing, Vienna, Austria. URL <https://www.R-project.org/>
