# Supplementary material for: Seeing for speaking: Semantic and lexical information provided by briefly presented, naturalistic action scenes
Source: PLoS One. 2018 Apr 13;13(4):e0194762. doi: 10.1371/journal.pone.0194762 (PMC5898714; doi:10.1371/journal.pone.0194762)

**Figures A - E illustrating interactions between Prime Conditions and Repetition, for Experiment 1a, 1b, 1c and Experiment 2 and Experiment 3**

**Figure A: Experiment 1A: Interaction Condition x Repetition**
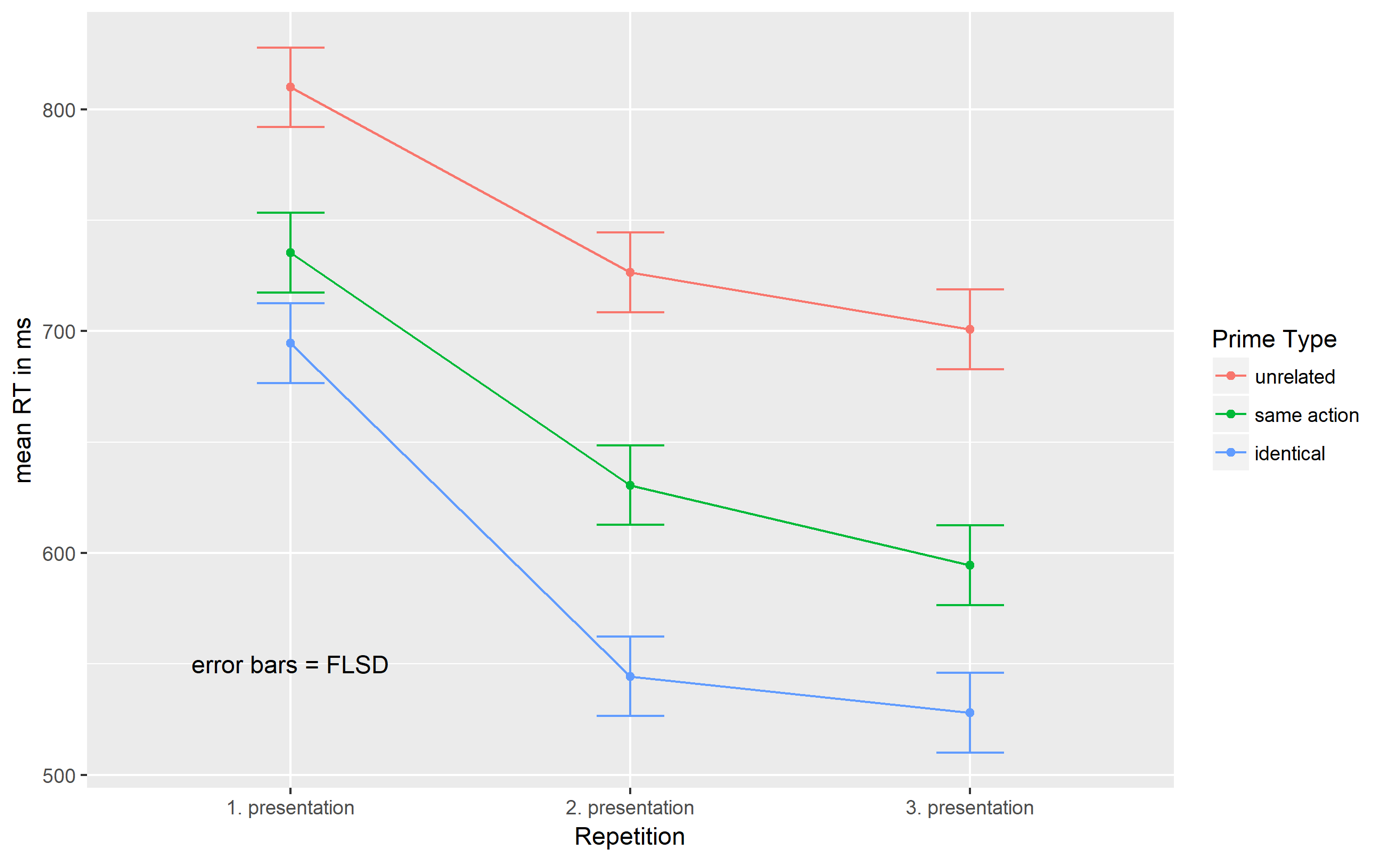


**Figure B: Experiment 1B: Interaction Condition x Repetition**


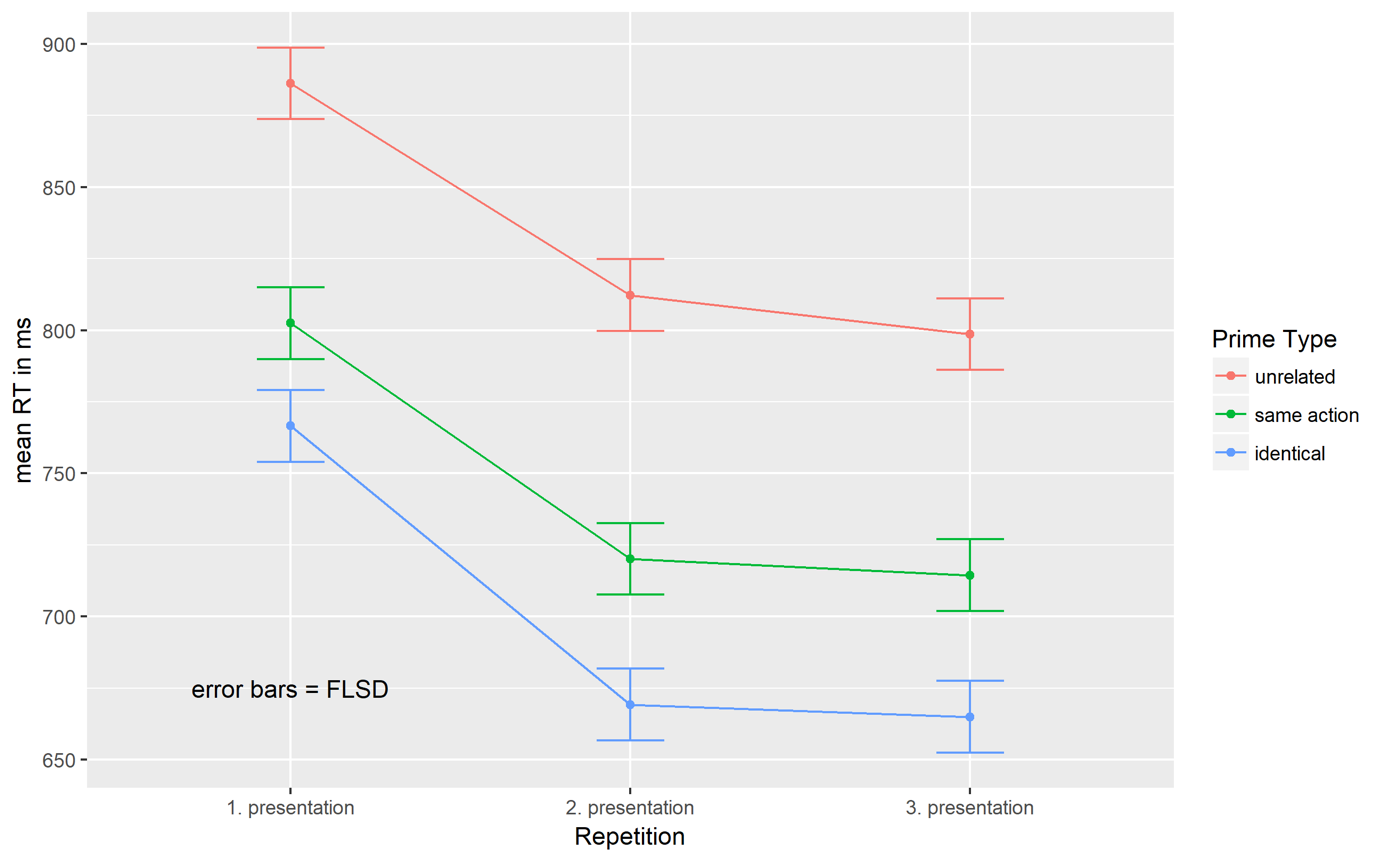


**Figure C: Experiment 1C: Interaction Condition x Repetition**
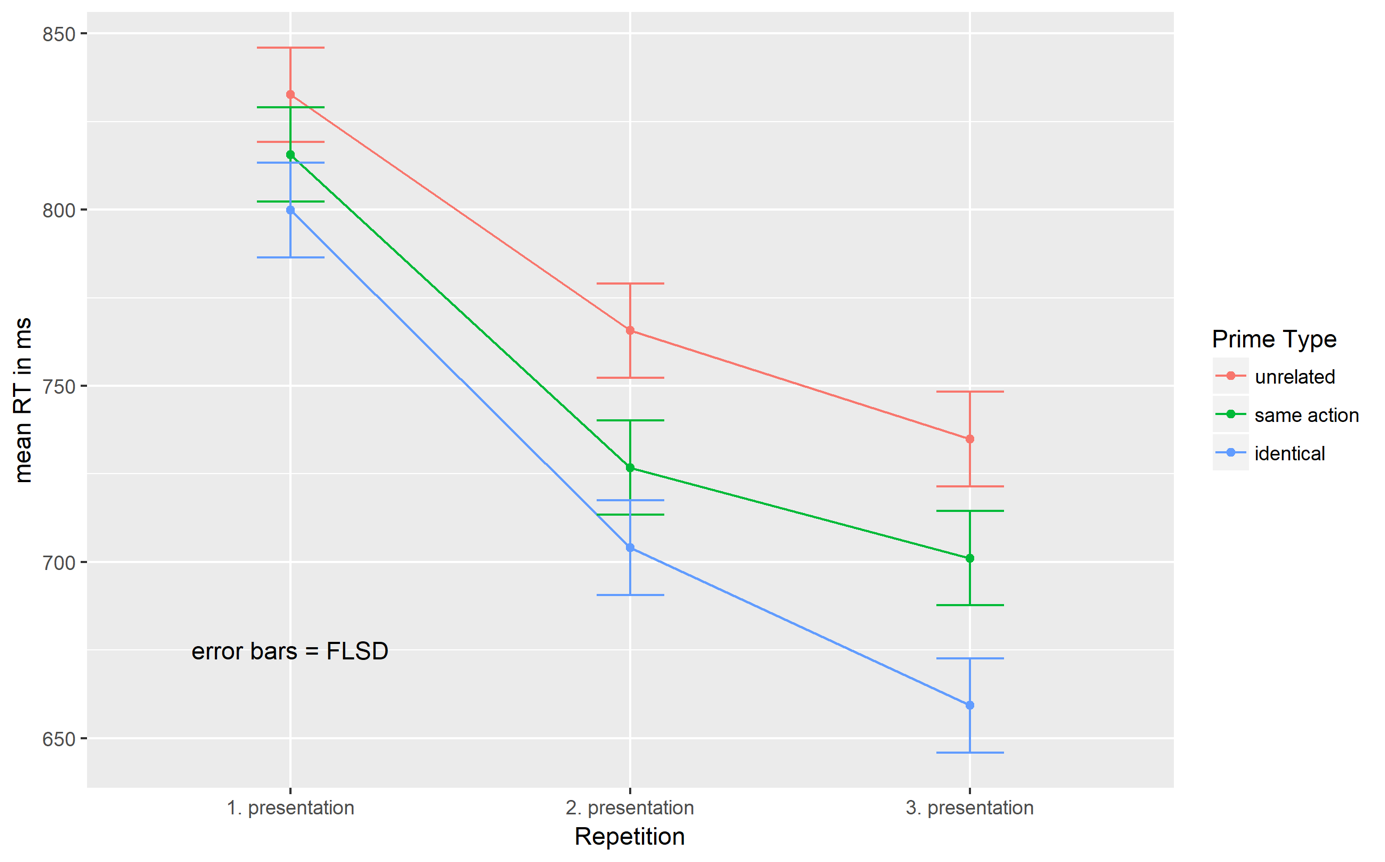


**Figure D: Experiment 2: Interaction Condition x Repetition**
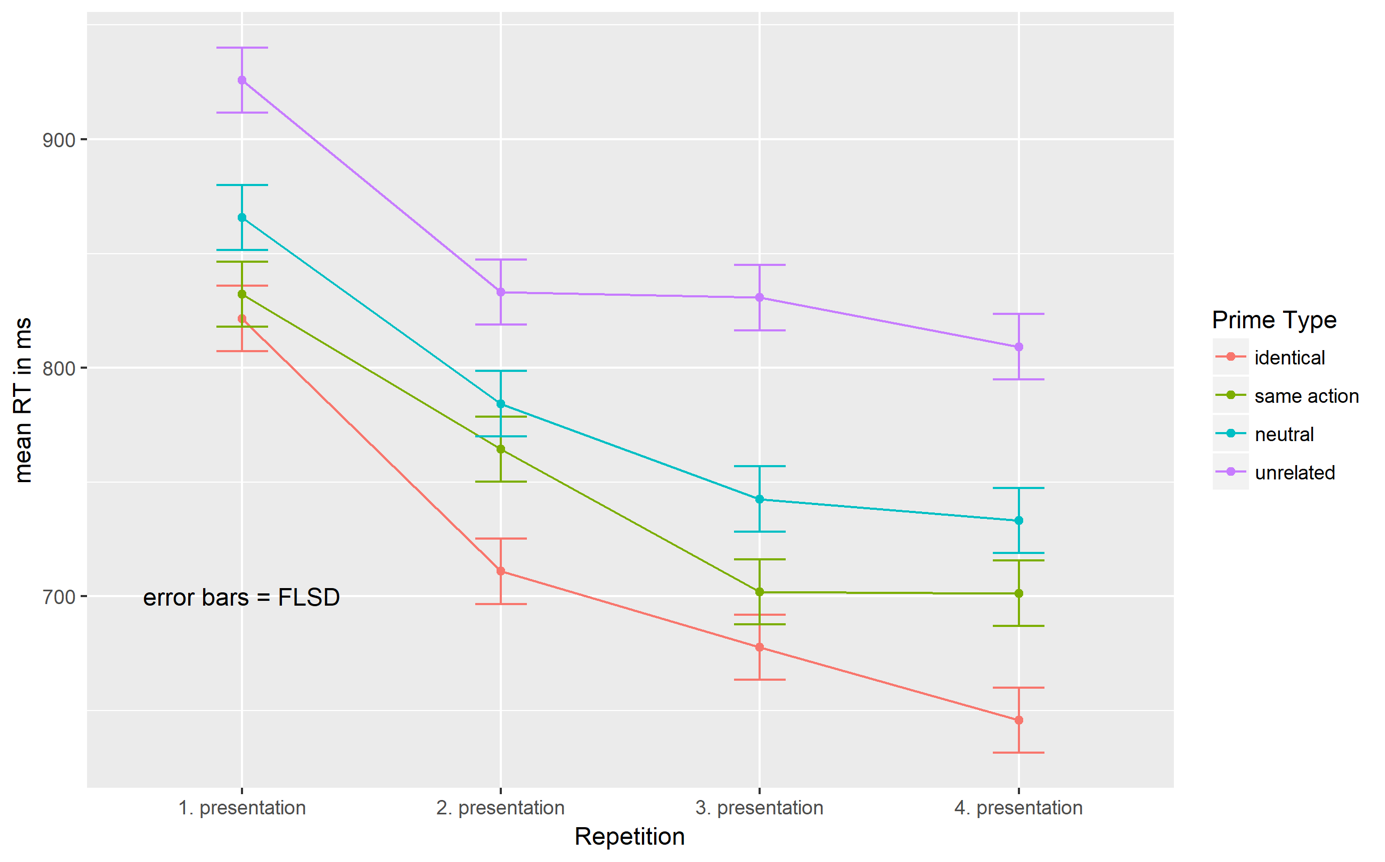


**Figure E: Experiment 3: non-significant Interaction Prime Type (Form, Identical) x Relatedness x Repetition (=prime repetition)**
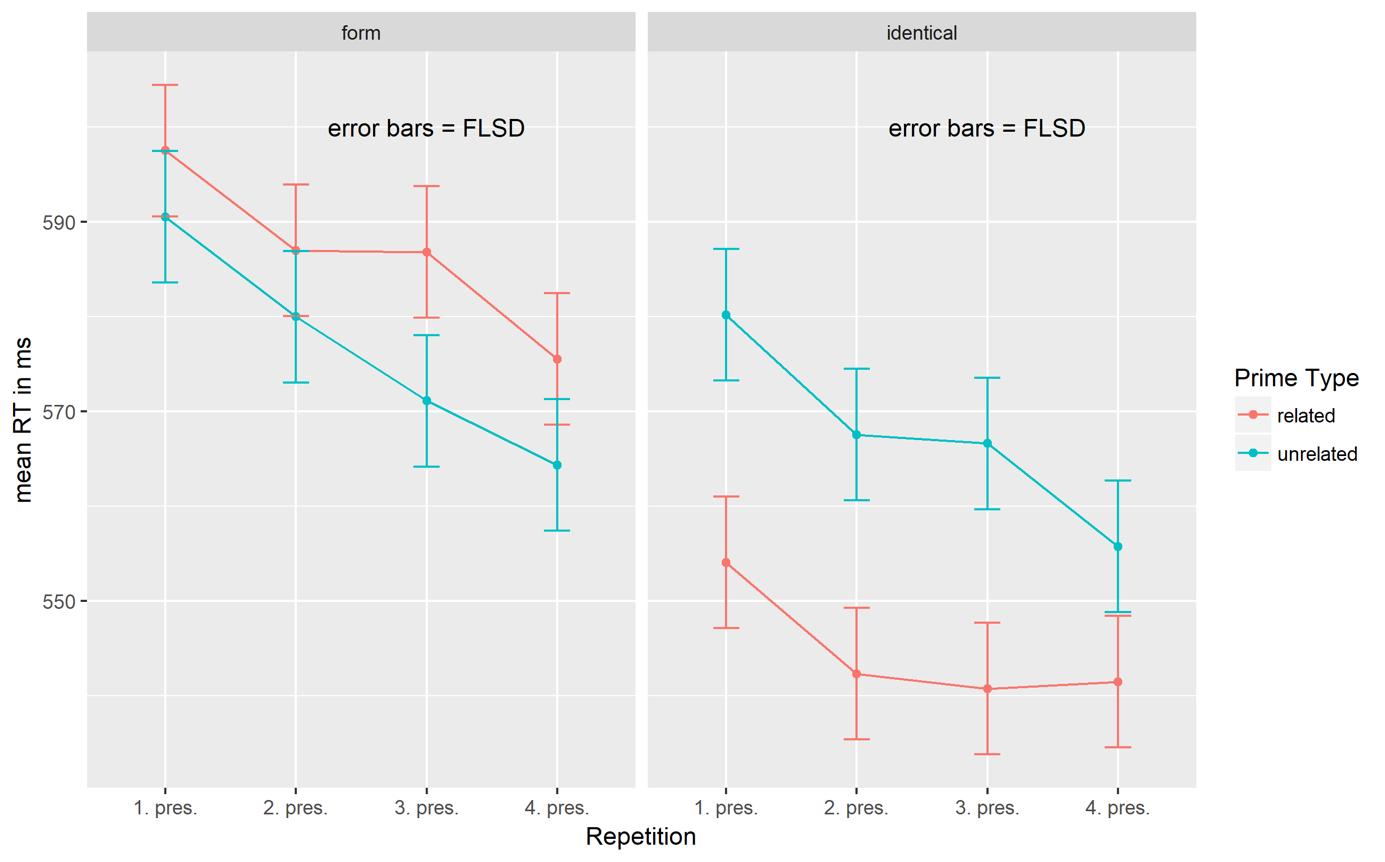

Supplement: S2 File — Figures A—E illustrating interactions for all experiments. (DOCX) [file pone.0194762.s002.docx]
